# Onecut-dependent Nkx6.2 transcription factor expression is required for proper formation and activity of spinal locomotor circuits

**DOI:** 10.1038/s41598-020-57945-4

**Published:** 2020-01-22

**Authors:** Mathilde Toch, Audrey Harris, Olivier Schakman, Elena Kondratskaya, Jean-Luc Boulland, Nicolas Dauguet, Stéphanie Debrulle, Charlotte Baudouin, Maria Hidalgo-Figueroa, Xiuqian Mu, Alexander Gow, Joel C. Glover, Fadel Tissir, Frédéric Clotman

**Affiliations:** 1 Université catholique de Louvain, Institute of Neuroscience, Laboratory of Neural Differentiation, Brussels, Belgium; 2 Université catholique de Louvain, Institute of Neuroscience, Laboratory of Cell Physiology, Brussels, Belgium; 3 Laboratory for Neural Development and Optical Recording (NDEVOR), Section for Physiology, Department of Molecular Medicine, Institute of Basic Medical Sciences, University of Oslo, Oslo, Norway; 4 Norwegian Center for Stem Cell Research, Department of Immunology and Transfusion Medicine, Oslo University Hospital, Oslo, Norway; 5 Université catholique de Louvain, de Duve Institute, Flow cytometry and cell sorting facility (CYTF), Brussels, Belgium; 6 Department of Ophthalmology/Ross Eye Institute, New York State Center of Excellence in Bioinformatics and Life Sciences, University at Buffalo, Buffalo, NY 14203 USA; 7 Wayne state University, Center for Molecular Medicine and Genetics, Carman and Ann Adams Department of Pediatrics, Department of Neurology, Detroit, Michigan USA; 8 Université catholique de Louvain, Institute of Neuroscience, Laboratory of Developmental Neurobiology, Brussels, Belgium; 9 Present Address: CIBER de Salud Mental (CIBERSAM), Madrid, Spain; 10 Present Address: University of Cadiz, Cadiz, Spain

**Keywords:** Developmental neurogenesis, Cell type diversity, Neuronal development, Neural circuits

## Abstract

In the developing spinal cord, Onecut transcription factors control the diversification of motor neurons into distinct neuronal subsets by ensuring the maintenance of *Isl1* expression during differentiation. However, other genes downstream of the Onecut proteins and involved in motor neuron diversification have remained unidentified. In the present study, we generated conditional mutant embryos carrying specific inactivation of *Onecut* genes in the developing motor neurons, performed RNA-sequencing to identify factors downstream of Onecut proteins in this neuron population, and employed additional transgenic mouse models to assess the role of one specific *Onecut*-downstream target, the transcription factor Nkx6.2. *Nkx6.2* expression was up-regulated in Onecut-deficient motor neurons, but strongly downregulated in Onecut-deficient V2a interneurons, indicating an opposite regulation of *Nkx6.2* by Onecut factors in distinct spinal neuron populations. *Nkx6.2*-null embryos, neonates and adult mice exhibited alterations of locomotor pattern and spinal locomotor network activity, likely resulting from defective survival of a subset of limb-innervating motor neurons and abnormal migration of V2a interneurons. Taken together, our results indicate that Nkx6.2 regulates the development of spinal neuronal populations and the formation of the spinal locomotor circuits downstream of the Onecut transcription factors.

## Introduction

Movement is the principle expressive output of the central nervous system. Locomotor movements are initiated in the brain either cortically or subcortically, but are expressed by channeling activity to locomotor networks, located primarily in the ventral part of the spinal cord, that include rhythm-generating circuits called Central Pattern Generators (CPGs). The spinal locomotor networks ensure the integration of the multiple inputs that regulate motor activity and control the rhythm, speed, and coordination of the locomotor movements. CPGs are composed of interconnected spinal interneurons (INs) of diverse types that generate patterned activity that is then imposed on the motor neurons (MNs) that innervate skeletal muscles. Although many regulatory genes that direct the differentiation of these spinal neuron populations have been identified and the role of some of these genes has been extensively studied^1^, the complete genetic programs underlying the specification of the various neuron types within locomotor networks are far from fully deciphered.

During embryonic development, spinal MNs diversify into different subsets characterized by distinct molecular identities, locations, and synaptic connections. At the thoracic level, visceral MNs that innervate sympathetic neurons of the paravertebral ganglia are clustered in the preganglionic column (PGC) whereas somatic MNs that innervate dorsal axial muscles or body wall muscles are clustered in the median motor column (MMC) and the hypaxial motor column (HMC), respectively. At brachial and lumbar levels, MNs that innervate the ventral or dorsal regions of the limbs are clustered in the medial and lateral portions of the lateral motor column (LMCm and LMCl), respectively^2^. LMC MNs further diversify into motor pools, each innervating a single muscle^3,4^. MN diversification is controlled by a dynamic network of transcriptional regulators that include Onecut (OC) factors. OC factors, namely OC-1 (also called HNF-6 for Hepatocyte Nuclear Factor-6), OC-2 and OC-3, are transcriptional activators present in the digestive tract and the CNS during development^5–9^. In neural tissue, they regulate the production^10^, diversification^11–13^, distribution^10,12–15^ or maintenance^10,15,16^ of specific neuronal populations as well as the assembly of neuromuscular junctions^17^. In the spinal cord, OC factors are expressed in newly born MNs^18^, and control the diversification of MNs by directly regulating the expression of the *Isl1* gene^11^. Furthermore, OC factors contribute to the progression of MN differentiation by recruiting MN-promoting transcriptional complexes to specific enhancer elements^19,20^. In addition, OC proteins have recently been shown to participate in the control of V2a IN diversification and migration, and to regulate in these neurons the expression of *Pou2f2*^13^. However, other genes acting downstream of OC factors in the control of MN or IN development remain unknown.

To identify genes downstream of OC factors in MNs, we generated conditional *Oc* mutant embryos wherein *Oc* genes are specifically inactivated in MNs, and we compared the transcriptome of control and of conditional *Oc* mutant MNs using RNA sequencing (RNA-seq). Here, we show that lack of *Oc* expression in MNs leads to upregulation of *Nkx6.2* expression in the LMC region. Surprisingly, by analyzing *Oc* constitutive mutants, we find that lack of OC factors in the spinal cord additionally downregulates *Nkx6.2* expression in the V2a INs, indicating opposite effects of OC factors on *Nkx6.2* in these two spinal neuron types. This suggests that Nkx6.2 acts downstream of OC factors in neuron populations that participate in the locomotor network, prompting us to test the requirement of Nkx6.2 for proper motor behavior including locomotion. Adult *Nkx6.2*-null mice exhibited some general motor deficits and specific locomotion defects characterized by altered coordination, abnormal stepping and fatigability, and *Nkx6.2*-null neonates exhibited shorter cycle periods and impaired alternation during non-weight-bearing locomotion (swimming). Analysis of fictive locomotion in *Nkx6.2*-null neonates demonstrated specific defects in CPG output. In the embryonic spinal cord of *Nkx6.2*-null mice, we observed a decrease in the number of LMCl MNs and alterations in the distribution of V2a INs. Adult *Nkx6.2*-null mice exhibited muscular hypertrophy in the portion of the quadriceps normally innervated by Nkx6.2^+^ MNs. Altogether, these results indicate that Nkx6.2 is regulated by OC factors in the embryonic spinal cord and is required for normal development of spinal locomotor and other motor circuits.

## Materials and methods

### Ethics statement and mouse lines

All experiments were performed strictly in accordance with the European Community Council directive of 24 November 1986 (86–609/ECC) and the decree of 20 October 1987 (87–848/EEC). Mice were raised in our animal facilities and treated according to the principles of laboratory animal care, and experiments and mouse housing were approved by the Animal Welfare Committee of Université catholique de Louvain (Permit Number: 2013/UCL/MD/11 and 2017/UCL/MD/008). The morning on which a vaginal plug was detected was defined as embryonic day (e) 0.5. A minimum of three embryos (n ≥ 3) of the same genotype was analyzed in each experiment. The embryos were harvested at embryonic days (e)10.5, e12.5 or e14.5 depending on the mouse line, and staging was confirmed using conventional staging criteria. The *Oc1*^*flox/flox*^;*Oc2* ^*flox/flox*^^16,21^ mutant mice were crossed with *Rosa26R-YFP/Olig2-Cre*^22^ transgenic mice bearing heterozygous-null mutations for *Oc1* and *Oc2* genes (Rosa26-YFP;*Olig2Cre;Oc1*^*+/−*^*;Oc2*^*+/−*^), to obtain conditional double knockout of *Oc1* and *Oc2* in MNs. The combined inactivation of *Oc1* and *Oc2* in MNs completely abolished the expression of *Oc3*^10,11,15^. *Nkx6.2*^*+/LacZ*^ mouse embryonic stem cells^23^ were used to generate *Nkx6.2* mutant mice (*Nkx6.2*^*−/−*^). To promote *LacZ* expression, *Nkx6.2* mice were crossed with PGK-Cre mice to remove the neomycin resistance cassette flanked by LoxP sites located downstream of the *LacZ* sequence.

### FACS, RNA purification, and RNA-sequencing

Spinal cords from e10.5 control or cdKO mice were harvested and dissociated using a neural tissue dissociation kit (MACS; Miltenyi Biotec #130-092-628) according to the manufacturer instructions. Dissociated cells were sorted by FACS (BD FACSAria III) to collect YFP-positive cells. Sorted cells were collected in TRIzol reagent, and RNA was purified with the Rneasy micro kit (QIAGEN #74004). RNA concentration and quality were assessed using a Bioanalyzer (Agilent) and submitted to Genewiz to prepare an ultra-low input RNA-seq library before sequencing with an Illumina HiSeq. Preliminary data were analyzed by Genewiz using the standard RNA-seq data analysis package. RNAseq data have been deposited in the GEO repository (accession number: GSE141949).

### *In situ* hybridization (ISH) and immunofluorescence

For ISH, collected embryos were immersion-fixed in ice-cold 4% paraformaldehyde (PFA) in phosphate buffered-saline (PBS) overnight at 4 °C, washed thrice in PBS for 10 minutes, incubated in PBS/30% sucrose solution overnight at 4 °C, and embedded and frozen in PBS/15% sucrose/7.5% gelatin. Fourteen-μm sections were prepared, and ISH was performed as previously described with DIG-conjugated Nkx6.2 antisense RNA probes (primer pair: 5′ GCTAAAAAGAAGCAAGACTCGG 3′ and 5′ CTCCGACGAGGACGTGTTAAA 3′).

For immunofluorescence, collected embryos were immersion-fixed in 4% PFA/PBS for 15, 25 or 35 minutes at 4 °C according to their embryonic stage, and processed as for ISH. Immunolabeling was performed on fourteen-μm serial cryosections as previously described^18^. Primary antibodies against the following proteins were used: BetaGal (chicken; 1:2000; Abcam #ab9361), Chx10 (sheep; 1:500; Exalpha Biologicals #X1179P), Er81 (rabbit; 1:10000; Covance), Foxp1 (goat; 1:1000; R&D Systems #AF4534), Gata3 (rat; 1:50; Absea Biotechnology #111214D02), GFP (chick; 1:1000; Aves Lab #GFP-1020), OC-1 (guinea pig; 1:2000^15^; or rabbit; 1:100; Santa Cruz #sc-13050; or sheep; 1:1000 R&D Systems #AF6277), Isl1 (goat; 1:3000; Neuromics #GT15051), Lhx3 (mouse; 1:1000, DSHB #67.4E12), MafA (guinea pig; 1:500; kindly provided by T. Müller), cMaf (rabbit; 1:5000; kindly provided by H. Wende), Nkx6.1 (mouse; 1:2000; DSHB #F55A10), nNOS (rabbit, 1:4000; Immunostar #24287), OC-2 (rat; 1:400^24^; or sheep; 1:500; R&D Systems #AF6294), OC-3 (guinea pig; 1:6000), Olig2 (rabbit; 1:2000; Millipore #AB9610), RALDH2 (rabbit; 1:10000) and Shox2 (mouse; 1:500; Abcam #AB55740). The secondary antibodies donkey anti-chicken/AlexaFluor 488, anti-guinea pig/AlexaFluor 594 or 647, anti-mouse/AlexaFluor 594 or 647, anti-rabbit/AlexaFluor 488, 594 or 647, anti-rat/AlexaFluor 488 or 647, anti-sheep/AlexaFluor 594 or 647 and goat anti-mouse IgG1 specific/AlexaFluor 594, purchased from ThermoFisher Scientific or Jackson Laboratories, were used at 1:2000 or 1:1000 dilution, respectively.

Immunofluorescence and ISH images from cryosections were acquired on an EVOS FL Auto Imaging System (ThermoFisher Scientific) or a confocal laser Scanning biological microscope FV1000 Fluoview using FV10-ASW 01.02 software (Olympus). The images were processed using Adobe Photoshop CS5 software to match brightness and contrast with the observations. For each embryo (n ≥ 3), neuron counts from one side of a minimum of five spinal cord sections at brachial, thoracic or lumbar levels were obtained using the count analysis tool of Adobe Photoshop CS5 software. Raw data were exported from Adobe Photoshop CS5 software to Sigma Plotv12.3 software to perform statistical analyses, and histograms were generated using Microsoft Excel. Appropriate statistical tests were applied depending on the number of comparisons and the data distribution and variance in each experimental group. For analysis of cell counts based on comparisons of two groups (control versus mutant), standard Student’s or Welch’s t-tests or Mann-Whitney U-tests were performed. Quantitative differences were considered significant at p < 0.05. Quantitative analyses of V2a IN spatial distributions were performed as previously described^12^. Briefly, in a transverse section of the spinal cord, height (*H*) was defined as the distance from the ventral limit of the central canal to the dorsal-most edge of the spinal cord, and width (*W*) as the distance from the central canal to the most lateral edge. For each V2a IN, distance (dIN) and angle (αIN) were measured from the ventral limit of the central canal to the IN soma using the ruler analysis tool in Adobe Photoshop CS5 software. Relative dorso-ventral (DV) and medio-lateral (ML) positions of V2a INs were expressed as percentages of spinal cord height and hemicord width respectively: DV position and ML position were defined as (dIN ∗ sin αIN)/*H* and (dIN ∗ cos αIN)/*W*, respectively (adapted from^25^), and ML versus DV values were plotted using Matlab software R2013a (Mathworks, Canada). Statistical analyses of ventral IN distribution were performed using a two-sample Hotelling’s T2 test, which is a two-dimensional generalization of the Student’s t-test. The analysis was implemented using the NCSS software package.

### Histological analyses of hindlimb muscles

Quadriceps and gastrocnemius muscles were dissected at 2.5 or 5 months of age and weighed. They were immersion-fixed in 4% PFA for 72 hours, paraffin-embedded and sectioned transversely at 20 micrometers. Sections were deparaffinized in Toluene 100% for 3x5 minutes, immersed in isopropanol 100% for 30 seconds and then rehydrated in a reverse ethanol series (100 to 30%, 2 min for each step). Sections were histologically stained with hemalun/eosin or labeled with Wheat Germ Agglutinine (WGA)/rhodamine (rabbit; 1:150; Vector RL-1022; Laboconsult) diluted in PBS and applied for 2 hours at RT. After washes in PBS, the slides were mounted as described above. Images were acquired with a Slide Scanner (3DHistech Pannoramic P250 Flash III) using CaseViewer software (3DHistech Ltd.) software. Central nuclei were counted on the entire RF portion of the quadriceps on at least 3 sections for each genotype (n = 3). For each muscle (n = 3), muscle fiber area was measured for at least 50 fibers in 5 sections using ImageJ software Raw data were exported to Microsoft Excel to draw the histograms.

### Behavioral tests in adult mice

Behavioral tests were performed on male heterozygote *Nkx6.2*^+/−^ (as control) or *Nkx6.2*-null mutant mice, starting at two months of age. All mice were handled and trained for at least two weeks before starting experiments, and weighed each experimental day. The rotarod test assesses the ability of mice to avoid falling from a rotating rod, and thus tests a conglomerate of motor skills including grip, balance and coordination. The Runway test evaluates the ability of mice to locomote across an elevated narrow runway towards their home cage. These two tests were performed as previously described^14^. The Catwalk test assesses gait during locomotion and was performed as previously described^17^. The wire test assesses grip fatigability and was performed as previously described^26^.

### Behavioral tests in neonatal mice

The vestibulospinal reflex test specifically assesses the vestibulospinal reflex and was performed as previously described^27^ on P4 control or *Nkx6.2-null* mice. For each mouse, the test was performed ten times while video recording at 100 frames/sec. For the analysis, we wrote a script in ImageJ to compare the position of the hindlimb at the start of the rotation and at the end of the reflexive extension (see Supplemental Fig. 1). If no extension occured, the end-point measurement was made at 200 ms. The script extracts the starting image (just before the rotation starts) and the image that the observer defines as the maximal hindlimb extension (or the image at 200 ms if no extension). The last image is then rotated 90^o^ counterclockwise to be in the same orientation as the starting image. A transparency overlay is created and the last image is manually re-aligned to the first. Lastly, the observer manually measures the distance between the paws of the overlaid images. The swimming test permits characterization of locomotor movements under non-weight-bearing conditions. The test was performed on P4 control or *Nkx6.2-null* mice. Briefly, the mouse pups were first habituated to the swimming chamber, which consisted of a 28 cm diameter glass dish containing 22 ^o^C water. On the test day, each pup was placed in the chamber and free swimming was recorded for 20 sec with a NX3-S1 video camera (IDT, Tallahassee, FL, USA) fitted with a Canon EF-S 18–200 mm f/3.5–5.6 IS), using manufacturer-supplied software (Motion Studio) at 100 Hz. The pup was then removed from the chamber, dried, warmed and returned to the litter. For analysis, a 10 sec period containing the best swimming performance (typically in the middle of the 20-sec session) was isolated and hindlimb tracking was performed using ImageJ- For each hindlimb, coordinates and timepoints were recorded at the moment of full flexion and full extension. Both the vestibulospinal reflex test and the free swimming test were done blinded to mouse genotype. All data were analyzed using ImageJ, and genotypes were disclosed at the end of the analysis.

### Fictive locomotion analysis in neonatal mice

Neonatal mice (P3-P4) were deeply anesthetized with an overdose of Isoflurane, decapitated, and the entire spinal cord isolated in ice-cold oxygenated low Ca^2+^ artificial cerebrospinal fluid (ACSF, in mM: 128 NaCl, 4 KCl, 1 CaCl_2_, 1 MgSO_4_, 0.5 NaH_2_PO_4_, 21 NaHCO_3_, 30 D-glucose) as previously described^28,29^. The preparation was transferred to a recording chamber and continuously superfused thereafter with oxygenated normal-Ca^2+^ ASCF (in mM: 2 CaCl_2_) at a rate of 2–5 ml/min at ambient temperature (23–25 ^o^C). The preparation was left to rest for 30 min before recording. The electrophysiologist was blinded to mouse genotype until data analyses were completed.

Ventral root (L2 and L5) potentials were recorded using DPA differential amplifiers (NPI electronics, Germany), in AC-mode with bandpass filtering at 100 Hz − 1.7 kHz, digitized at 10 kHz (PicoScope 5442 A, Pico Technology) and registered with a Picoscope 6 (Pico Technology, Cambrigeshire, UK) as previously described^28,29^. L2 and L5 ventral root discharges correspond predominantly to activity in hindlimb flexor and extensor MNs, respectively^30,31^. Alternating discharge in left versus right and L2 versus L5 ipsilateral ventral roots was interpreted to indicate locomotor CPG activity. Fictive locomotion (FL) was triggered by bath application of neurotransmitters /agonists, N-methyl-D-aspartic acid (NMDA, 5 µM), serotonin (5-hydroxytryptamine creatinine sulfate, or 5-HT, 10 µM), and dopamine (DA, 50 µM), as previously described^28,29,32,33^. Stock solutions of NMDA, serotonin, and dopamine were prepared fresh daily. At least 30 min of recording was performed for each preparation after drug application, with the last 10–15 min of recording used for the analysis of FL after rhythm stabilization. FL was assessed in Nkx6.2−/− and age-matched control preparations.

To analyze FL discharge patterns, a custom algorithm of quasi-periodic oscillation analysis was used as previously described^28,29^. Traces were filtered, rectified, resampled and smoothed using a weighted central moving average method (Hamming function) in Sigview (SignalLab) and SciDAVis (open-source and cross-platform, http://scidavis.sourceforge.net/). This represented bursting discharges as quasi-periodic wave oscillation events. The primary analysis of these oscillation events was performed using Clampfit 10.2 software (Axon Instruments) and parameters such as burst duration and cycle period were determined. Rhythm and inter-root phase relationships were further analyzed using two different methods: (1) circular analysis^30^, to obtain a statistical measure of L2-L5 coordination, and (2) quantification of phase relationships between flexor and extensor activity by cross-correlation analysis. The rhythmicity of bursts was assessed by autocorrelation analysis as previously described^29,31^. Finally, the discharge frequencies of each individual root were analyzed. Five-fifteen min time intervals were used to plot frequency distribution graphs (normalized to peak value, bean distribution fitted by Kernel smooth fit).

### Statistical testing

Statistical tests were applied based on the number of comparisons and the data distribution and variance in each group. Unless indicated elsewhere, for analysis based on comparisons of two groups (control or mutant), standard Student’s t-tests or Mann-Whitney U tests were performed. Differences were considered significant at p < 0.05. To compare frequency distributions, the Kolmogorov-Smirnov two sample non-parametric test for empirical distributions was used.

## Results

### Generation of MN-specific *Oc*-deficient embryos

**Figure 1:**
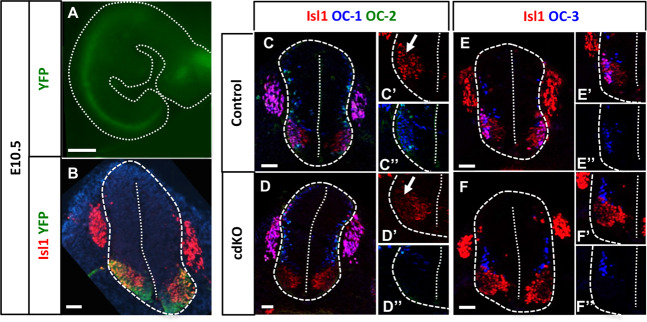
Validation of the conditional motor neuron-specific *Oc*-null mice. (**A**) YFP-positive cells in a whole *Olig2-Cre/Rosa26-YFP/Oc1*^*Δ/−*^*Oc2*^*Δ/−*^ embryo at e10.5, viewed from right side. The dotted line delineates the embryo, YFP fluorescence is observed along the spinal cord and in the encephalon. Scale bar = 1000 μm. (**B**) Immunostaining for YFP and Isl1 on a transverse section of control embryo at e10.5. Differentiating Isl1-positive MNs contained YFP. YFP fluorescence was also detected in cells ventral to MNs, likely corresponding to V3 INs. (**C-F**) Immunostaining for Isl1, OC-1, OC-2 and OC-3 at e10.5 in control or conditional double-mutant (*Oc1*^*−/−*^*Oc2*^*−/−*^) embryos. OC factor expression was lost in Isl1-positive MNs and *Isl1* expression was decreased in the most laterally situated MNs (arrows). Scale bars = 20 μm.

To identify genes downstream of OC factors possibly involved in MN diversification, we generated conditional mutant mice wherein *Oc* genes are specifically inactivated and the YFP fluorescent reporter is simultaneously expressed in MNs (Fig. 1A) (*Olig2-Cre/Rosa26-YFP/Oc1*^*Δ/−*^*Oc2*^*Δ/−*^ mice, referred to hereafter as cdKO mice). As previously described^10–12,15^, the deletion of *Oc1* and *Oc2* abrogated the expression of *Oc3*, enabling us to study the consequences of the absence of all 3 OC factors from MNs. We first evaluated the efficacy and specificity of the Cre recombinase to inactivate *Oc* genes in MNs. At e10.5, a majority of YFP-positive cells contained Isl1, a marker of spinal MNs. However, YFP was also detected in a neuron population ventral to MNs, corresponding to V3 INs (Fig. 1B), likely stemming from transient expression of *Olig2* in p3 progenitors during ventral spinal cord patterning^34^. In control embryos, OC factors were produced as soon as progenitors exited the cell cycle and their expression pattern overlapped in most of the newly born MNs (Fig. 1C,E)^18^. In cdKO mice, OC-1, OC-2 (Fig. 1D-D’’) and OC-3 (Fig. 1F-F’’) were lost in Isl1-positive MNs. Furthermore, Isl1 production was strongly decreased in the differentiating MNs located laterally, as previously demonstrated in constitutive *Oc* mutant mice (arrows in Fig. 1C’,D’)^11^. Thus, the *Olig2-Cre* allele efficiently eliminates the expression of *Oc* factors in MNs.

### *Nkx6.2* expression is differentially regulated by OC factors in MNs and INs

In an effort to identify genes downstream of OC factors during MN differentiation, we performed an RNA-sequencing comparison between control and cdKO YFP-positive cells at e10.5 (GEO repository accession number: GSE141949). Among genes showing a differential expression level (Supplemental Table 1) and compared to previous microarray experiments in e11.5 constitutive *Oc* mutant spinal cord^13^, the expression of *Nkx6.2* was upregulated (1.32-fold increase) in cdKO YFP-positive cells as compared to controls, whereas it was downregulated (1.42-fold decrease) in the spinal cord of constitutive *Oc* mutant embryos^13^. Nkx6.2 (previously named Gtx) is a transcription factor involved in the ventral patterning of the embryonic spinal cord. In the p1 progenitor domain, Nkx6.2 represses *Dbx1* expression and thereby allows the generation of V1 INs^35–37^. *Nkx6.2* is also expressed in myelinating oligodendrocytes and in a specific motor pool of the lumbar LMC^23,38^.

**Figure 2:**
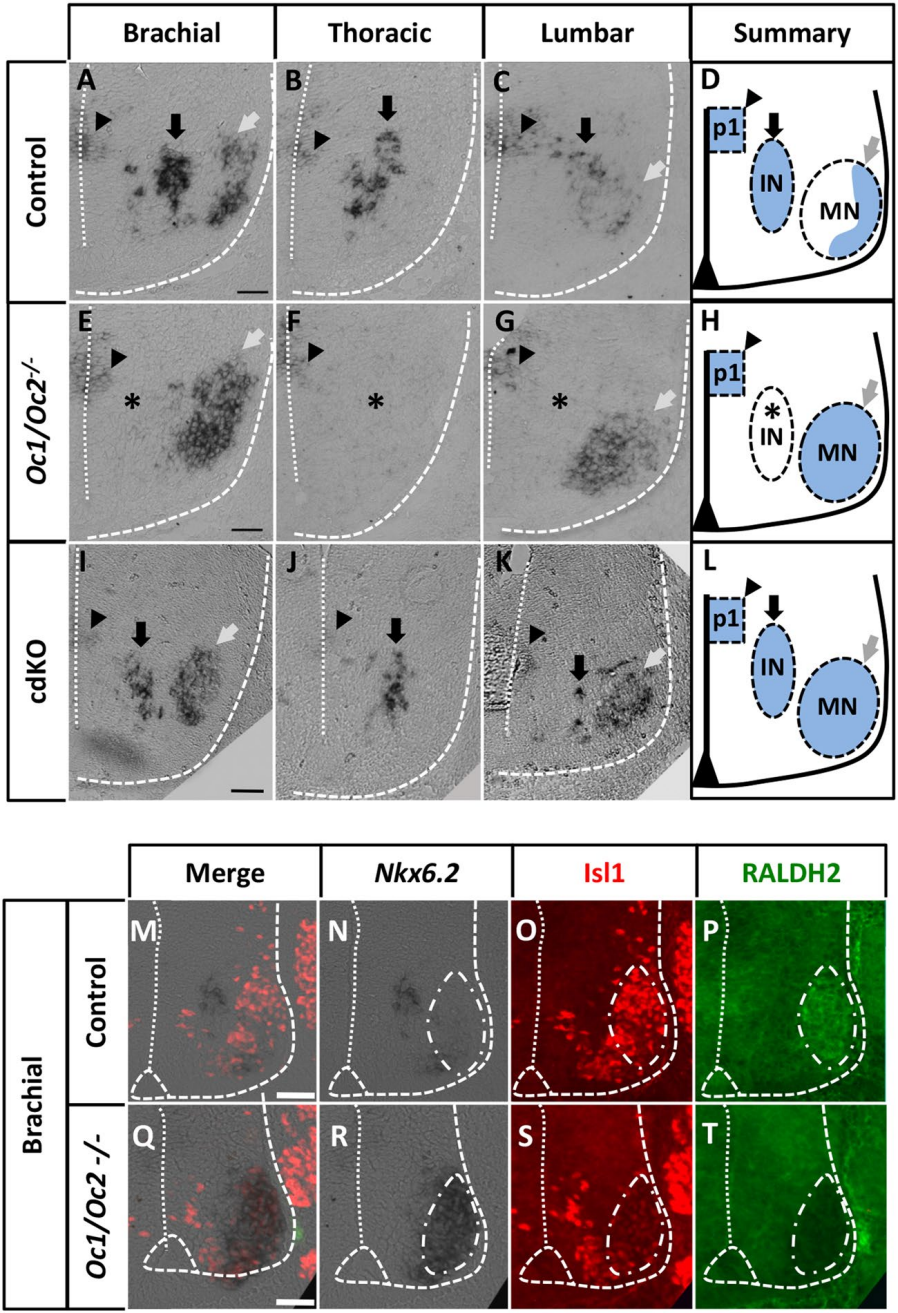
OC factors inversely regulate the expression of *Nkx6.2* in motor neurons and in ventral interneurons. **(A-I)** *In situ* hybridization for *Nkx6.2* on transverse sections at brachial, thoracic and lumbar levels of control or *Oc* constitutive double-mutant spinal cord at e11.5 or of conditional (cdKO) *Oc* mutant spinal cord at e12.5. (**D,H,L**) shows a summary of *Nkx6.2* expression (blue) in each genotype. (**A-D**) In control embryos, *Nkx6.2* was expressed in p1 progenitors (arrowheads) and in ventral INs (black arrows) at all 3 levels, and in some MNs (grey arrows) at brachial and lumbar levels. (**E-H**) In the constitutive mutant, *Nkx6.2* was present in the p1 domain, lost in ventral INs (asterisks), but expanded in the brachial and lumbar MNs (grey arrows). (**I-L**) In the conditional mutant, *Nkx6.2* was expressed in the p1 progenitors (arrowheads) and in ventral INs (black arrows), and expanded in brachial and especially lumbar MNs (grey arrows). (**M-T**) To determine if this expansion of *Nkx6.2* expression was specific to MNs, we combined *in situ* hybridization for *Nkx6.2* (Q-T) with immunofluorescence labeling for Isl1 (U-X) and RALDH2 (Y-BB) on transverse spinal cord sections at the brachial level of control or constitutive mutant at e12.5. RALDH2 labels all the limb-innervating LMC MNs. The *Nkx6.2 in situ* hybridization signal coincides with, and masks, RALDH2 immunostaining (which defines LMC MNs), indicating that *Nkx6.2* expression in the *Oc* mutant embryos expanded to all the LMC cells. Scale bars = 100 μm.

To confirm the data from RNA-seq and microarray experiments and to understand the opposite regulation of *Nkx6.2* in constitutive and conditional *Oc* mutants, we performed *in situ* hybridization for *Nkx6.2* on transverse sections of control, constitutive (e11.5) or conditional (e12.5) *Oc* mutant spinal cords. In control embryos, *Nkx6.2* transcripts were detected in the p1 domain (arrowheads in Fig. 2A-D, Fig. 2M-N) and ventral INs (black arrows in Fig. 2A-D, Fig. 2M-N) at brachial, thoracic and lumbar levels of the spinal cord, and in some MNs at brachial and lumbar levels (grey arrows in Fig. 2A–D, Fig. 2M,N). Previous analyses showed that the number of MNs in the LMC columns and of ventral INs in each cardinal population is unchanged in the absence of OC factors^11,13^. In constitutive *Oc* mutant (*Oc1/Oc2*^*−/−*^) embryos, *Nkx6.2* expression was unchanged in the p1 domain but was lost in ventral INs (asterisks in Fig. 2E–H) and expanded to include more brachial and lumbar MNs (grey arrows in Fig. 2E,G,H). By contrast, in cdKO embryos, *Nkx6.2* expression was unchanged in the p1 domain and in the ventral INs (arrowheads and black arrows in Fig. 2I–L), but expanded in brachial and lumbar MNs similar to the constitutive mutants (grey arrows in Fig. 2I,K,L). Thus, the consequences of the MN-specific inactivation of *Oc* genes on *Nkx6.2* expression appeared to be specific to limb-innervating MNs, although the number of LMC neurons is not changed in the absence of OC factors^11^. To confirm that this expansion of *Nkx6.2* expression was specific to LMC MNs, we combined at e12.5 *in situ* hybridization for *Nkx6.2* (Fig. 2M–T) with immunofluorescence for the MN marker Isl1 (Fig. 2O,S) and for RALDH2, which is specifically expressed in LMC neurons^39,40^ (Fig. 2P,T). In mutant embryos, *Nkx6.2* expression was coincident with, and masked, the RALDH2 immunolabeling (Fig. 2T), suggesting that *Nkx6.2* was expressed within RALDH2-positive cells corresponding to LMC MNs. Taken together, these observations indicate that the dowregulation of *Nkx6.2* expression in the constitutive mutant is due to elimination of expression in ventral INs despite expansion in LMC MNs^11,13^, whereas the upregulation seen in the cdKO mutant is due to expansion in LMC MNs without loss in ventral INs.

### Absence of *Nkx6.2* results in general motor deficits and abnormal locomotion

**Figure 3:**
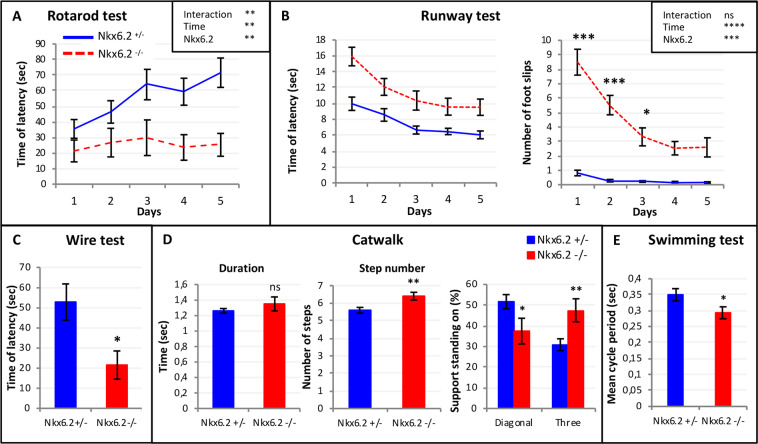
Motor deficits and abnormal locomotion in *Nkx6.2*-null mutant adult mice. **(A)** Behavioral tests were performed on *Nkx6.2*-null mice (Nkx6.2−/−) and compared to *Nkx6.2* heterozygoous control mice (*Nkx6.2*^*+/−*^) as controls. The rotarod test was performed on five successive days. *Nkx6.2*-null mice (n = 14) were unable to stay on the rotating rod as long as heterozygous control mice (n = 18), and did not improve over time, as did the control mice. Genotype, time and interaction of both showed significant differences on the duration. (**B**) The Runway test was performed with heterozygous control (n = 18) or *Nkx6.2*-null (n = 14) mice for five successive days, and traversal time and number of foot slips were recorded. *Nkx6.2*-null mice traversed the runway more slowly and with more foot slips than control mice, although both groups improved over successive days. Genotype and time showed significant differences on the duration. (**C**) The wire test was used to test grip fatiguability. *Nkx6.2*-null mice were more fatigable than control mice (control: n = 18 vs mutant: n = 14; p = 0.014). (**D**) Locomotion was tested on the catwalk. Locomotion in *Nkx6.2*-null mice (n = 10) was characterized by an increased number of steps during the same period of time compared to control mice (n = 15), without any change in the speed of forward motion (6.4 ± 0.22 steps vs 5.6 ± 0.16 steps in control mice; p = 0.007). i.e. an increase in gait frequency and decrease in stride length. *Nkx6.2*-null mice also exhibited a decrease in the percentage of time with simultaneous contact with the substrate by diagonal paws (p = 0.040) and an increase in the percentage of time of simultaneous contact by three paws (p = 0.008), indicating a change in gait pattern. (**E**) To assess non-weight-bearing locomotion, the swimming test was performed on P4 *Nkx6.2*-null mice (n = 17) with heterozygote littermates as controls (n = 11). *Nkx6.2*-null neonates had shorter mean cycle times (p < 0.05).

Adult *Nkx6.2*-null mice exhibit motor coordination defects, which have been attributed to defective myelination in the CNS^23^, but more specific motor skills including locomotion were not investigated. To assess a possible requirement of *Nkx6.2* expression for proper locomotion, we first reevaluated some general motor skills and then studied specific features of locomotion in adult *Nkx6.2*-null mice, compared to littermates that were heterozygous for the *Nkx6.2* mutation (control mice). Control mice initially remained more than 35 s on the rotating rod, and this duration increased with repetition, indicative of learning. In contrast, *Nkx6.2*-null mice were not able to remain on the rotating rod more than 20 s on average, and no improvement was observed with time (Fig. 3A). Using the Runway test, control mice traversed the beam in 10 s on average with virtually no foot slips, and performance improved with repetition. In contrast, *Nkx6.2*-null mice were slower and exhibited many foot slips, although both of these deficits improved substantially with repetition (Fig. 3B). Finally, the wire test revealed that *Nkx6.2*-null mice were more fatigable than control mice (Fig. 3C; p = 0.014). Thus, consistent with a previous report^23^, *Nkx6.2*-null mice exhibited several general motor and motor learning deficits.

To assess whether specific locomotion-related abilities were altered, gait parameters were analyzed using the Catwalk assay. The duration to cross the catwalk was not different between control and *Nkx6.2*-null mice (Fig. 3D), indicating that the forward motion speed was normal. However, mutants used a larger number of steps (6.4 ± 0.22 steps vs 5.6 ± 0.16 steps in control mice; p = 0.007) during the same period of time (Fig. 3D), indicative of a higher gait frequency and a shorter stride length. Furthermore, *Nkx6.2*-null mice had fewer instances of simultaneous contacts with diagonal pairs of paws (p = 0.04) and more instances of simultaneous contacts with three paws (p = 0.008) (Fig. 3D), indicative of a change in gait pattern.

To assess non-weight-bearing locomotion, and to bridge the behavioral studies of locomotion to the neonatal stages used for electrophysiological studies (see below), we assessed swimming in P4 *Nkx6.2*-null mice with heterozygote littermates as controls. *Nkx6.2*-null neonates had on average shorter mean cycle periods (Fig. 3E; p < 0.05), in line with the increased gait frequency in adult *Nkx6.2*-null mice. We also noted that some *Nkx6.2*-null neonates, but no control mice, exhibited a locomotor pattern in which alternation was largely absent, with movements of fore- and hindlimbs on the same side dominating. This prompted us to examine the relationship between mean cycle period and deficient alternation more closely. We found that within the *Nkx6.2-*null group, the alternation-deficient locomotor pattern was exhibited by the *Nkx6.2-*null mice with the shortest mean cycle periods (p = 0.001). Moreover, comparison to the control group showed that the ratio of missed alternations to mean cycle period was significantly higher in the *Nkx6.2-*null group (p = 0.037). Taken together, these data indicate that, in addition to general motor defects, *Nkx6.2-*null mice display altered locomotion characterized by increased step frequency and eroded locomotor pattern.

### *Nkx6.2*-null neonates exhibit normal fictive locomotor patterning but altered cycle frequency distribution

Perturbed locomotor activity in adult and neonatal *Nkx6.2*-null mice notwithstanding, our behavioral observations do not pinpoint the locus of locomotor deficits. For example, they cannot formally exclude the possibility that the defective locomotor phenotype in *Nkx6.2*-null mice arises secondarily to myelination defects. To address this question, we assessed fictive locomotion (FL) on isolated *Nkx6.2*-null and heterozygous littermate control spinal cords, in which the locomotor CPG can be assessed directly, and at P3-P4, when central myelination is at an early stage^23,41^. There was no difference between control and *Nkx6.2*-null newborn mice in the vestibulospinal reflex test at P3-P4 (Supplemental Fig. 1), indicating no effect of the mutation on this descending pathway and suggesting no general central conduction velocity differences between mutants and controls at this neonatal stage.

**Figure 4:**
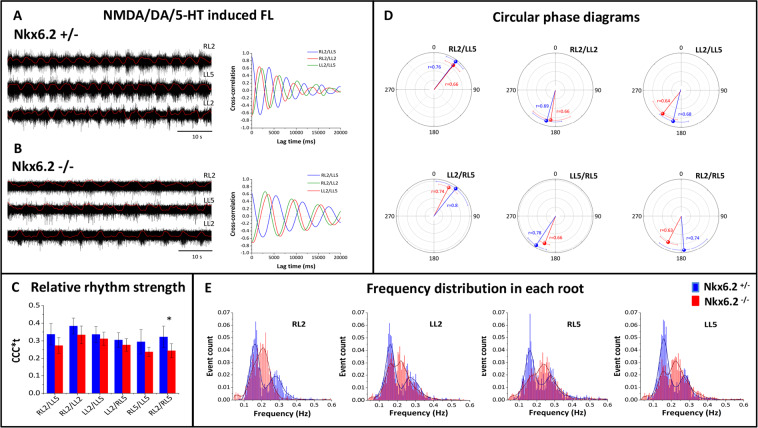
Fictive locomotion in control or *Nkx6.2* mutant neonates. (**A,B**) Raw traces of fictive locomotion pattern in ventral roots (RL2, LL5, LL2) recorded in *Nkx6.2*^*+/−*^ (heterozygous control, **A**) or *Nkx6.2*-null (**B**) spinal cord preparations (right panel). Red trace represents rectified and smoothened version of the original trace (black). Cross-correlograms for corresponding root pairs are shown in the right panels. Time bar represents 10 s. (**C**) Cross-correlation coefficient integral on time for all root pairs analyzed in *Nkx6.2*^*+/−*^ (heterozygous control) or *Nkx6.2*-null preparations. (**D**) Circular phase diagrams show coordination between root pairs in *Nkx6.2*^*+/−*^ (heterozygous control, blue circles and error bars) and *Nkx6.2*-null (red circles and error bars) preparations. (**E**). Distributions of burst frequencies during fictive locomotion in each root (RL2, LL2, RL5, LL5) in *Nkx6.2*^*+/−*^ (blue) and *Nkx6.2*-null (red) preparations.

FL was elicited by bath application of 5-HT/NMDA/DA (10 μM, 5 μM and 50 μM respectively), and recorded via the right (R) and left (L) L2 or L5 ventral roots. The typical pattern of FL with left/right and flexor/extensor alternation (RL2/LL2, LL2/LL5) and synchronous discharges for RL2/LL5 ventral roots was recorded in preparations of both genotypes (control n = 8, *Nkx6.2*-null n = 11; example of original traces for Nkx6.2^+/−^ and *Nkx6.2*-null shown in Fig. 4A,B). Cross-correlation analysis showed proper phase relationships in *Nkx6.2*-null preparations (Fig. 4B). Additionally, the cross-correlation coefficient (CCC) integrated over time was calculated to estimate rhythm strength for each root pair. As shown in Fig. 4C, there was no statistical difference in CCC analyzed for 3 of the pairs of roots between control and *Nkx6.2*-null preparations, the exception being the RL2/RL5 root pair (p = 0.05, Mann Whitney U-test).

Next, we examined the average phase relationships for different root pairs. As shown in Fig. 4D, there were no statistically significant differences between control and *Nkx6.2*-null preparations in the preferred phases for root pairs expected to be synchronous or alternating. For the synchronous pairs RL2/LL5 and LL2/RL5, mean preferred phases were respectively 37.2 ± 17.3° (control, n = 5) versus 37.9 ± 17.0° (*Nkx6.2*-null, n = 7) and 37.8 ± 17.2° (control, n = 4) versus 26.9 ± 18.3° (*Nkx6.2*-null, n = 7). For the alternating pairs RL2/LL2 and RL5/LL5, mean preferred phases were respectively 196.1 ± 27.5° (control, n = 7) versus 188.7 ± 37.3° (*Nkx6.2*-null, n = 7) and 212.6.1 ± 18.2° (control, n = 4) versus 201.2 ± 9.8° (for *Nkx6.2*-null, n = 8). For the ipsilateral alternating pairs LL2/LL5 and RL2/RL5, mean preferred phases were respectively 194.2 ± 27.0° (control, n = 6) versus 217.6 ± 30.3° (*Nkx6.2*-null, n = 7) and 175.3 ± 43.0° (control, n = 5) versus 206.0 ± 19.0° (*Nkx6.2*-null, n = 6). Although left-right and flexor-extensor alternation  was not affected in *Nkx6.2*-null preparations, extra burst activity was sometimes recorded in RL5 (less often in LL5) roots, which could lead to a phase drift for inter-segmental alternation in mutants (data not shown).

Finally, we analyzed the frequency distribution of bursts. As shown in Fig. 4E, in control preparations we observed two frequency peaks for each root, a slow frequency peak at about 0.16 Hz and a fast frequency peak at about 0.27 Hz. In contrast, in *Nkx6.2*-null preparations, we observed only a single, intermediate frequency peak at about 0.22 Hz, but in some cases with a lower frequency shoulder. The frequency distributions for the RL2 and LL2 roots differed significantly between *Nkx6.2*+/− and *Nkx6.2*-null preparations (Kolmogorov-Smirnov test, p < 0.001). Significance was not reached for the RL5 and LL5 roots (p = 0.159 and p = 0.160 respectively). We also pooled the peak frequencies for all roots in the two groups (n = 3754 for controls n = 4510 for mutants), and found a similar differential distribution, with one pronounced peak for *Nkx6.2*-null preparations and a bi–modal distribution for control preparations (Kolmogorov-Smirnov test, p < 0.001; Fig. 4D).

Taken together, these observations demonstrate that *Nkx6.2*-null spinal cord preparations can generate normally patterned FL, but tend to have intermediate burst frequencies relative to heterozygous preparations. This might be related to the observation that heterozygous adult and neonatal mice have lower average gait frequencies and swim cycle frequencies than their *Nkx6.2*-null littermates.

### Absence of *Nkx6.2* results in muscle fiber hypertrophy in a portion of the quadriceps

**Figure 5:**
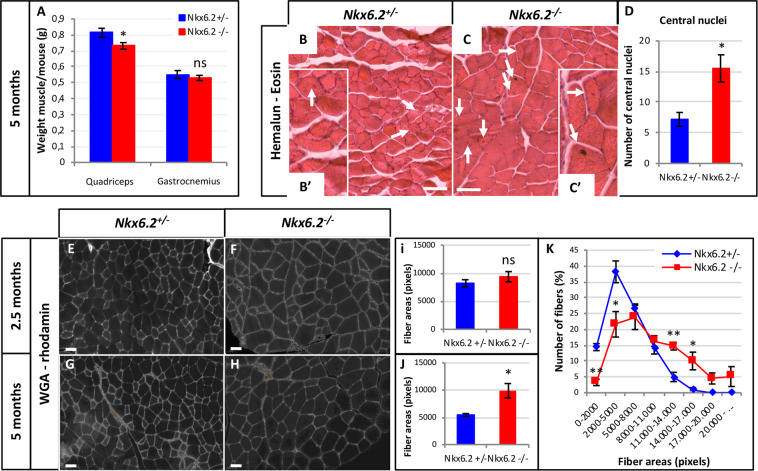
Absence of *Nkx6.2* results in Rectus Femoris muscle alterations. **(A)** Quadriceps and gastrocnemius muscles from *Nkx6.2* heterozygous (+/−) or null (−/−) mice at 5 months of age were weighted and normalized to the body weight. The weight of the quadriceps was lower in *Nkx6.2*-null mice than in heterozygous control mice (n = 7; p = 0.038), while the weight of the gastrocnemius muscle was unchanged. (**B-D**) Hemalun/eosin labelings of transverse sections in the Rectus Femoris portion of the quadriceps from heterozygous control or *Nkx6.2*^*-*^-null mice at five months of age. Insets show higher magnifications. The number of central nuclei (white arrows) was increased in the Rectus Femoris portion in the absence of *Nkx6.2* (n = 3; p = 0.016). Scale bar = 100 μm. (**E-J**) Wheat-Germ Agglutinin (WGA)/rhodamin labelings of transverse sections in the Rectus Femoris portion of the quadriceps muscle from heterozygous control or *Nkx6.2-null* mice at 2.5 or five months of age. Rectus Femoris fibers were larger in the absence of *Nkx6.2* at five months of age. Scale bar = 50 μm. (**K**) Distribution of fiber area in the Rectus Femoris portion of heterozygous control and mutant mice at 5 months of age. *Nkx6.2*-null mice exhibited a higher number of large diameter fibers compared to control mice (n = 3).

Altered formation or activity of spinal locomotor circuits often results in subsequent changes in innervation or morphology of the corresponding muscles. At hindlimb levels of the spinal cord, Nkx6.2 defines a specific motor pool supplying innervation to the *Rectus Femoris* (RF) portion of the quadriceps muscle (Fig. S2)^38^. Innervation of the RF was previously reported to be normal in *Nkx6.2*-null animals^38^, and we observed here that the density of neuro-muscular junctions was comparable in *Nkx6.2*-null mice and heterozygous control littermates (data not shown). In contrast, the weight of the quadriceps normalized to body weight was reduced in *Nkx6.2*-null mice compared to controls, whereas the weight of the gastrocnemius muscle was similar (Fig. 5A; n = 7; p = 0.038). In addition, the number of central muscle fiber nuclei in the RF portion of the quadriceps was increased in *Nkx6.2*-null mice (arrows in Fig. 5B–D; n = 3; p = 0.016), suggesting that it had undergone repetitive degeneration/regeneration cycles^42^. Furthermore, the mean cross-sectional area of RF fibers showed no change at 2.5 months of age (Fig. 5E,F,I) but an increase in *Nkx6.2*-null mice by 5 months of age (Fig. 5G,H,J n = 3; p = 0.03). Consistently, the distribution of fiber area at 5 months of age showed that RF portion of the quadriceps in *Nkx6.2*-null mice was composed of larger fibers than in control mice (Fig. 5K; n = 3). Thus, motor deficits in *Nkx6.2*-null mice are accompanied by changes in a specific hindlimb muscle, suggesting a specific effect on the respective MNs.

### *Nkx6.2* is necessary for proper maintenance of LMCl MNs at lumbar level

**Figure 6:**
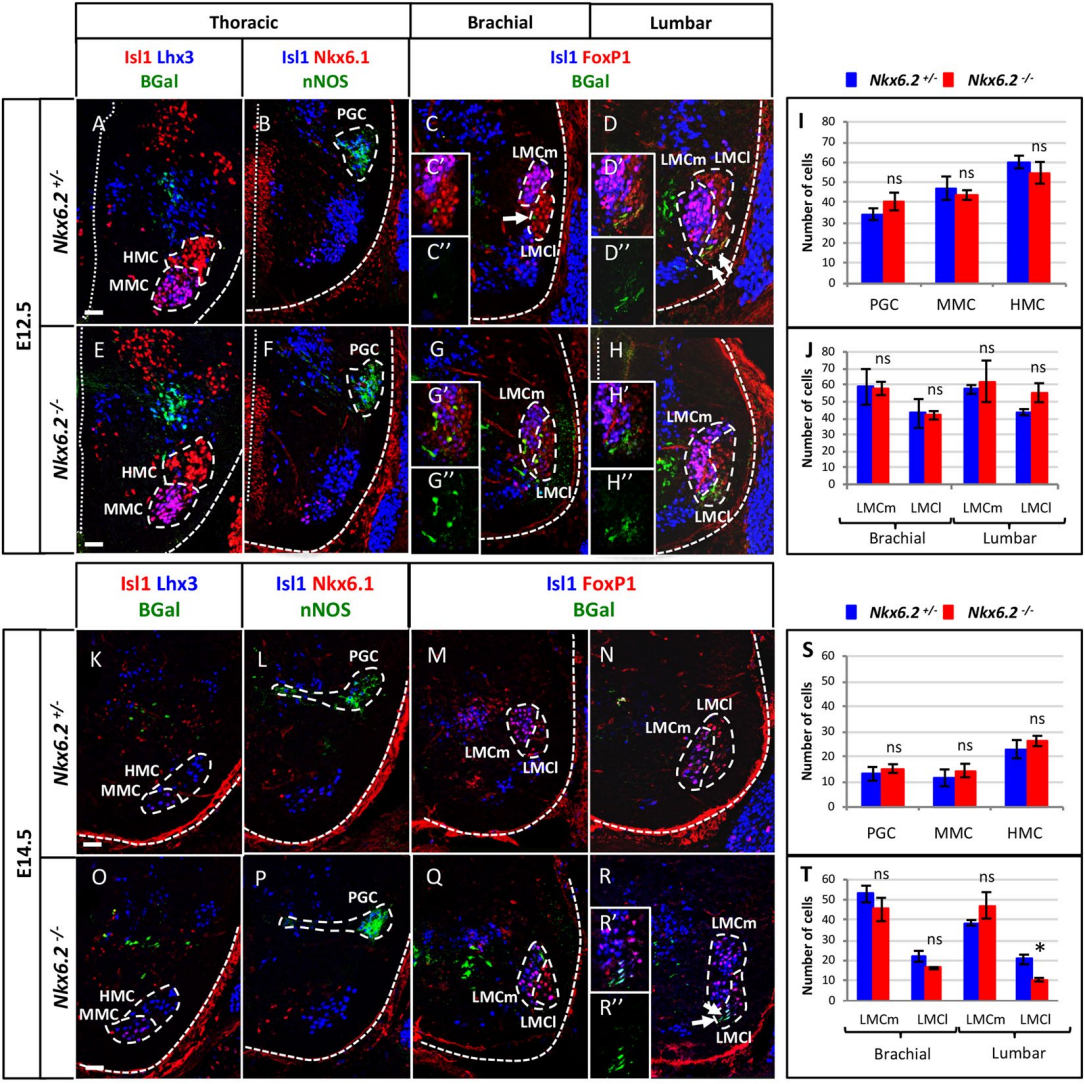
*Nkx6.2* is required for the maintenance of LMCl motor neurons. Immunostaining of transverse spinal cord sections of *Nkx6.2* heterozygous control or *Nkx6.2*-null mutant embryos at e12.5 (**A–J**) or e14.5 (**K–T**). (**A–J**) In the heterozygous control embryos at e12.5, β-galactosidase was absent from HMC (Isl1+), MMC (Isl1 + Lhx3 + ) and PGC (Isl1 + nNos + ) at thoracic level, but was detected in rare MNs of the LMCl (Foxp1 + Isl1-) at brachial level (arrow in **C**) and in a greater number of LMCl cells at lumbar level (arrows in **D**). In *Nkx6.2*-null mutants, the β-galactosidase signal was observed in more LMCl MNs at brachial (arrows in **G**) and at lumbar (arrows in **H**) levels. The production of HMC, MMC and PGC MNs was not affected in the absence of *Nkx6.2*. Similarly, no change was detected in the LMC at brachial or lumbar levels of the spinal cord. (**K-T**) At e14.5, β-galactosidase signal was only detected in some LMCl MNs at lumbar level (arrows in **R**) in *Nkx6.2* mutant mice. The production of HMC, MMC and PGC MNs at thoracic level was not changed by absence of *Nkx6.2*. In contrast, the number of LMCl MNs at lumbar level was significantly decreased in the *Nkx6.2*-null mice (**N,R,T**) with no perturbation of the LMC at brachial level and of the LMCm (Foxp1 + Isl1 + ) at lumbar level. n ≥ 3; * p < 0.05. Scale bar = 50 μm.

Although Nkx6.2 and its paralog factor Nkx6.1 are critical for ventral neural tube patterning, the absence of *Nkx6.2* in spinal progenitors is compensated by the presence of Nkx6.1. Therefore, *Nkx6.2* single mutants do not exhibit ventral patterning defects^35,43^, enabling us to study its role at later stages of spinal neuron development. Accordingly, we ascertained which neuron types depend on Nkx6.2 during later development, beginning with MNs. Previous studies have demonstrated *Nkx6.2* expression in MNs of the LMCl, which is characterized by expression of Foxp1 but not Isl1^38^. In the thoracic spinal cord of heterozygous *Nkx6.2*^*+/−*^ embryos at e12.5, β-galactosidase was absent from the somatic HMC and MMC MNs (Fig. 6A) as well as from the nNOS-positive visceral MNs (Fig. 6B), as expected^38^. At limb levels, although the β-galactosidase signal was weak, it was detected in a few MNs of the brachial LMCl (arrow in Fig. 6C), and a greater number of MNs in the most ventral part of the lumbar LMCl (arrows in Fig. 6D). In *Nkx6.2*-null embryos, a stronger β-galactosidase signal was detected in more of the brachial LMCl MNs. These β-galactosidase + MNs were interspersed with LMCm neurons (characterized by the expression of both Foxp1 and Isl1; arrows in Fig. 6G-G’’), consistent with a previous report showing that MNs expressing Nkx6 proteins are initially intermixed within the LMC before segregating into coherent clusters^38^. In the lumbar spinal cord of *Nkx6.2*-null mutants, β-galactosidase-positive MNs were located in the most ventral part of the LMCl (arrows in Fig. 6H-H’’) as in controls (Fig. 6D). At e14.5, the β-galactosidase signal was weaker and was only detected in some LMCl MNs in the lumbar *Nkx6.2*-null spinal cord (arrows in Fig. 6R). Thus, consistent with previous reports^38^, Nkx6.2 in differentiating MNs is restricted to a subset of LMCl MNs likely corresponding to a specific motor pool. The presence of β-galactosidase-positive MNs in the LMC of *Nkx6.2*-null spinal cords suggests that *Nkx6.2* expression is not required for the generation of the Nkx6.2 + MNs.

To evaluate if the absence of *Nkx6.2* impacts MN subsets, motor columns were studied at e12.5 (Fig. 6A–J) and e14.5 (Fig. 6K–T). At e12.5, no change was observed at thoracic levels in the number and location of MNs in the HMC (Isl1+), MMC (Isl1 + Lhx3+) (Fig. 6A,E,I) or PGC (Isl1 + nNOS+) (Fig. 6B,F,I), or at limb levels in LMCm (FoxP1 + Isl1+) or LMCl (FoxP1 + Isl1-) MNs (Fig. 6C,D,G,H,J). At e14.5, the developmental stage during which motor pools are specified, no change was observed in HMC, MMC (Fig. 6K,O,S) or PGC (Fig. 6L,P,S) MNs at thoracic levels or in either division of the LMC at brachial levels (Fig. 6M,Q,T). In contrast, the number of LMCl MNs was significantly decreased at lumbar levels in the *Nkx6.2*-null spinal cords (Fig. 6N,R,T; n = 3; p = 0.017). Taken together, these observations suggest that *Nkx6.2* expression is not required for the production of *Nkx6.2*-expressing LMC MNs, but is required for the survival of a discrete subset of LMCl cells.

To determine if the decrease in the number of LMCl MNs at lumbar level at e14.5 correlated with the absence of specific motor pools, the distribution of Nkx6.1 and Er81 was studied at e14.5. Nkx6.1, the paralog of Nkx6.2, is expressed in different motor pools within the LMCm whereas Er81, an ETS family protein, is expressed in motor pools within both divisions of the LMC^38,44^. Pea3, another ETS protein, has been shown to be co-expressed with Nkx6.2 within the LMCl^38^, but due to a lack of an effective Pea3 antibody, we were unable to characterize this MN subpopulation. Nonetheless, the number of Nkx6.1 + nor Er81 + MNs was unaltered in the lumbar LMC of *Nkx6.2*-null embryos (Fig. S3), suggesting that the absence of *Nkx6.2* in the LMCl does not impact on the generation or maintenance of Nkx6.1 or Er81 motor pools but results in the loss of another LMC neuron subset that remains to be identified.

### ***Nkx6.2*** regulates the distribution of V2a INs during spinal cord development

**Figure 7:**
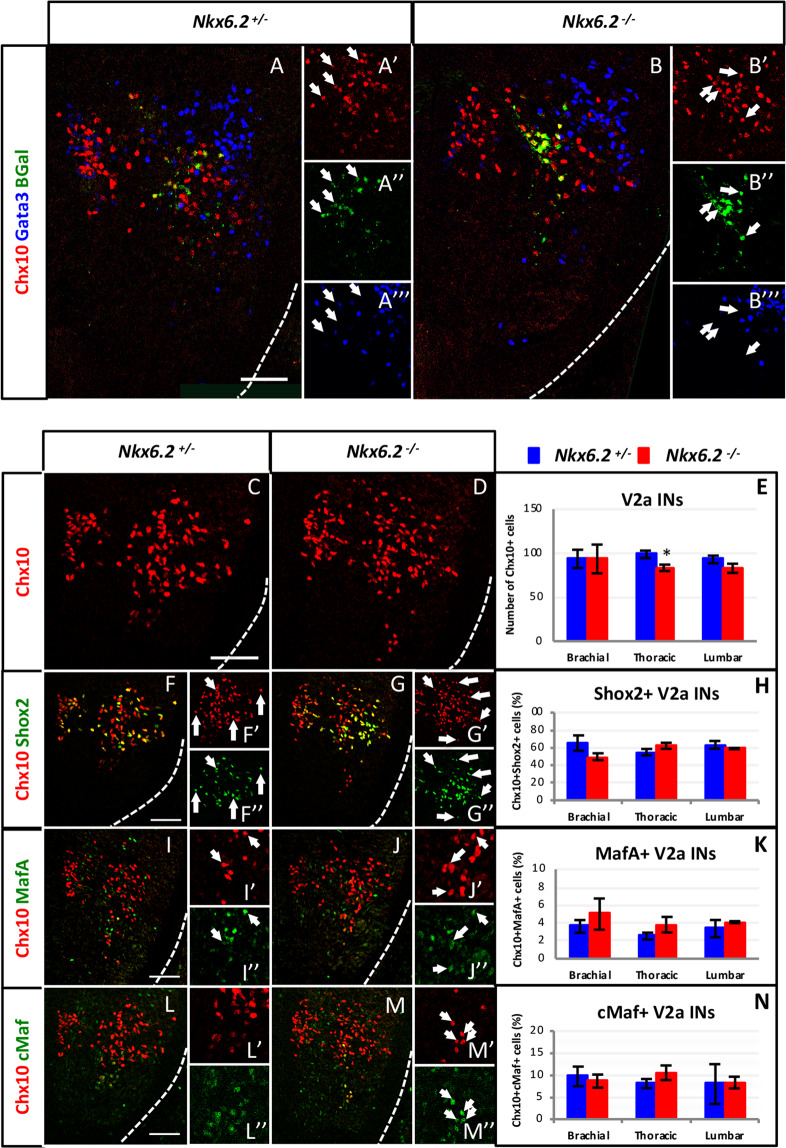
Nkx6.2 is not necessary for proper diversification of V2a interneurons. Immunostaining of transverse brachial spinal cord sections of *Nkx6.2* heterozygous control or *Nkx6.2*-null mutant embryos at e12.5. (**A)** In heterozygous control embryos, β-galactosidase was present in Chx10-positive cells corresponding to V2a INs (arrows), but was absent from Gata3 + V2b INs. (**B)** Similar observations were made in the *Nkx6.2*-null mutant embryos (arrows). (**C-E**) In the absence of Nkx6.2, the number of V2a INs was modestly decreased at the thoracic level, but not at the brachial or lumbar levels. (**F-N**) Among the examined V2a IN subpopulations, generation of the Shox2 + (**F-H**), MafA + (**I-K**) and cMaf + (**L-N**) subsets was unaffected. n = 3; * p < 0.05. Scale bars = 50 μm.

The deficits in motor behavior and changes in FL observed in *Nkx6.2*-null mice suggest that some aspects of the coordination of MN activity are altered. Therefore, we investigated further the role of Nkx6.2 in ventral IN development. In addition to MNs and the p1 domain^35,37^, *Nkx6.2* expression was detected in the V2 IN domain in the ventral spinal cord (Fig. 2A-D). To determine whether *Nkx6.2* is expressed in V2 INs, we compared the distribution of β-galactosidase with that of V2 markers in heterozygous *Nkx6.2*^*+/−*^ embryos at e12.5. Although the signal was weak, β-galactosidase was present in cells containing Chx10 corresponding to V2a INs (arrows in Fig. 7A). In contrast, β-galactosidase was never detected in Gata3 + V2b INs or in other IN populations (Fig. 7A and data not shown). In *Nkx6.2*-null embryos, the β-galactosidase signal was stronger and was co-detected with Chx10 in V2a INs (arrows in Fig. 7B), confirming expression of *Nkx6.2* in V2a postmitotic INs.

OC factors, which regulate the diversification and the distribution of V2a INs^13^, are thus required for *Nkx6.2* expression in V2a INs (Fig. 2E-H). To assess a possible function for Nkx6.2 in V2a development, we characterized the phenotype of V2a INs in *Nkx6.2*^−/−^ embryos at e12.5 (n = 3). In the absence of *Nkx6.2*, the total number of Chx10 + V2a INs was moderately decreased (16%) at the thoracic level, but was indistinguishable from controls at brachial or lumbar levels (Fig. 7C-E). Among the V2a subpopulations, none of the Shox2 + , MafA + or cMaf + subsets were altered in the absence of *Nkx6.2* (Fig. 7F-N). Hence, Nkx6.2 is not required for the diversification of the V2a INs but appears to be necessary for appropriate production of a subset of V2a INs at the thoracic level.

**Figure 8:**
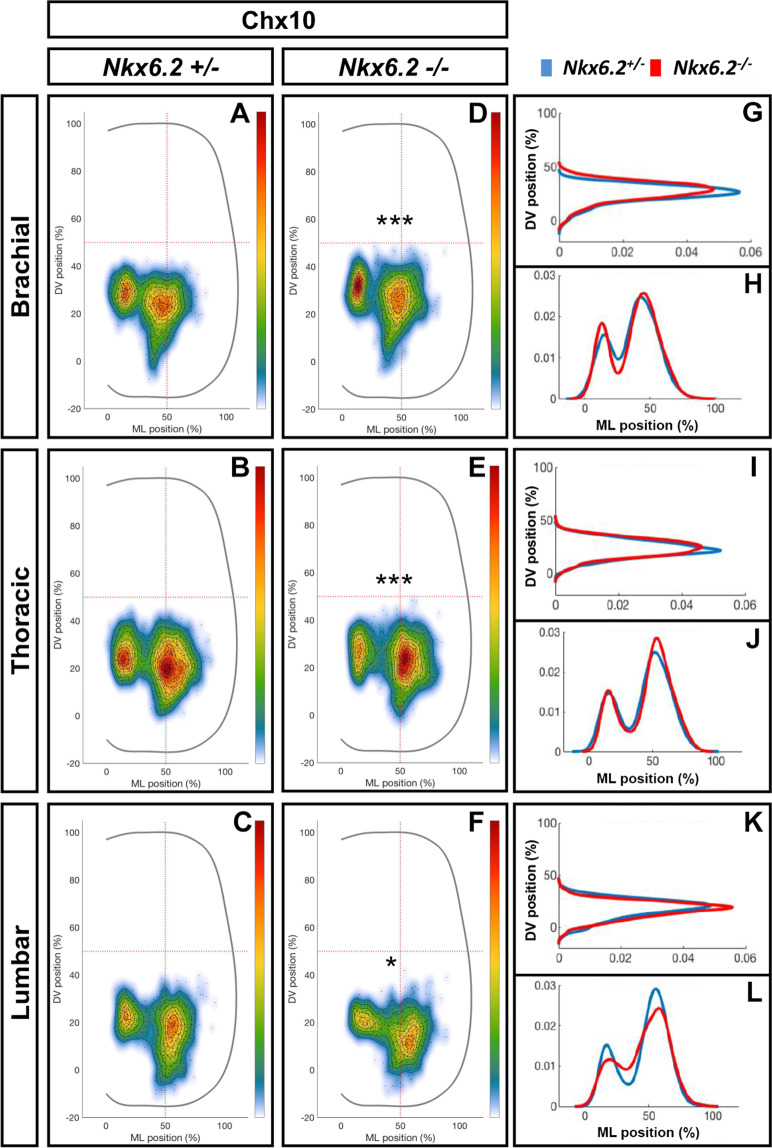
Nkx6.2 contributes to the regulation of the V2a interneuron distribution. Integrated distribution of V2a INs in the transverse plane of the spinal cord in *Nkx6.2* heterozygous control or *Nkx6.2*-null mutant embryos at e12.5 (only the right half of the spinal cord is shown). **(A-F)** Two-dimensional cell density heat-maps show the integration of V2a IN distribution from multiple sections from multiple embryos of each genotype. The color code represents cell density. **(G-L)** One-dimensional graphs (right) compare density distribution (arbitrary cell density units) in control (blue) and in mutant embryos (red) along the dorso-ventral (DV) or the medio-lateral (ML) axes of the spinal cord. (**A-C**) In *Nkx6.2* heterozygous control embryos, V2a INs are distributed in two connected clusters, a major central group and a minor medial group, at each level of the spinal cord. (**D-L)** In *Nkx6.2*-null embryos, the relative cell distribution between the two clusters is altered with greater separation of clusters and more cells in the medial group at the brachial level (**D, G-H**), more cells in the central group at the thoracic level (**E, I-J**), and fewer cells in less separated clusters at the lumbar level (**F, K-L**). n = 3; p < 0.001 at brachial and thoracic levels, p < 0.05 at lumbar level.

However, careful examination of the Chx10 immunostaining suggested that Nkx6.2 might contribute to the regulation of V2a IN position (Fig. 7C-D). Therefore, a quantitative comparison of the distribution of V2a INs was performed in control and in *Nkx6.2*-null mutants. In heterozygous *Nkx6.2*^*+/−*^ embryos, V2a INs were distributed between two interconnected clusters, a major central group, and a minor medial group, at each level of the spinal cord (Fig. 8A-C). In mutant embryos, the relative cell distribution between these clusters appeared altered (Fig. 8D-L). At brachial levels, the two clusters were more segregated with more cells in the medial group (Fig. 8D,G-H; n = 3; p < 0.001). At thoracic levels, the number of V2a INs was increased in the central group (Fig. 8E,I-J; n = 3; p < 0.001). Finally, at lumbar levels, the two clusters were less segregated than in control embryos (Fig. 8F,K-L; n = 3; p < 0.05). Taken together, these results demonstrate that Nkx6.2 is not required for the production of V2a INs but influences their ultimate distribution within the ventral part of the spinal cord.

## Discussion

**Figure 9:**
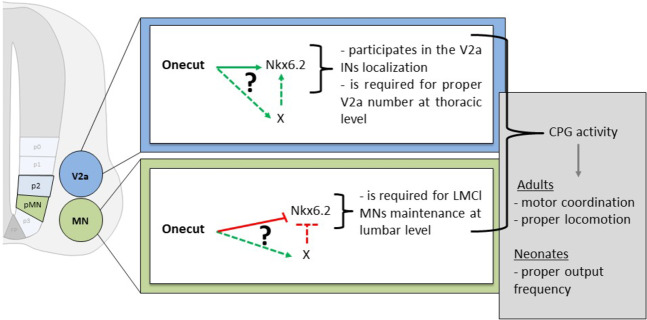
Working model for OC factor and Nkx6.2 contribution to CPG development. OC factors directly or indirectly stimulate *Nkx6.2* expression in V2a INs wherein Nkx6.2 regulate cell number and distribution. In contrast, OC factors directly or indirectly repress *Nkx6.2* expression in LMC MNs wherein Nkx6.2 is necessary to maintain the number of LMCl cells at lumbar level. The combined action of Nkx6.2 in V2a INs and MNs contribute to proper CPG formation and activity at both the neonatal and the adult age. Green: stimulation, red: inhibition, dotted lines: indirect regulations.

Several prior studies have identified a contribution of the OC factors to MN and IN development in the embryonic spinal cord^11–13,16,19,20^. Here, we show that OC factors differentially regulate the expression of *Nkx6.2* in V2a INs and MNs. We provide evidence that Nkx6.2 is required for proper motor function and locomotor behavior, and we demonstrate that Nkx6.2 is necessary for the survival of a subset of LMC MNs and for the proper distribution of V2a INs. We have thus identified Nkx6.2 as a novel regulator of spinal motor circuit formation downstream of the OC factors (Fig. 9).

Our observations indicate that OC transcription factors stimulate the expression of *Nkx6.2* in V2a INs while restricting it within LMC MNs. Consistently, results of a microarray experiment on constitutive *Oc*-null mutant spinal cord revealed that *Nkx6.2* was decreased in the absence of OC factors^13^, while RNA-seq comparison from OC-deficient MNs showed an increase in *Nkx6.2* expression. Three hypotheses could account for these observations. First, changes in the expression levels of *Nkx6.2* in specific spinal populations of *Oc* mutant embryos may be secondary to alterations in the identity or survival of these cells. In the absence of *Oc* factors, identity of the LMCm MNs is converted into that of the LMCl cells due to defective maintenance of *Isl1* expression, resulting in an expansion of LMCl MNs^11^. However, Nkx6.2 is restricted to a ventral pool of LMCl MNs in control embryos^38^ but is detected in all the LMC cells in *Oc*-deficient embryos. Conversely, *Nkx6.2* expression was strongly downregulated in V2a INs of *Oc* mutant embryos whereas the total number of V2a was unaffected^13^. Therefore, it seems unlikely that perturbations of *Nkx6.2* expression in the absence of OC factors may be secondary to differentiation or survival defects. Second, OC factors may regulate *Nkx6.2* in MNs and INs by different mechanisms. OC factors are described as transcriptional activators^45–48^. Thus, they could directly stimulate *Nkx6.2* expression in V2a INs, but indirectly downregulate its expression in MNs via a repression mechanism that remains to be identified. Identification of the OC factor binding sites in the regulatory sequences of *Nkx6.2* could address these possibilites. However, *Nkx6.2* regulatory sequences remain to be characterized and potential OC factor binding sites in the genome are common. Therefore, ChIP-seq experiments on isolated spinal neuron subsets would be necessary to identify the genes directly regulated by OC factors in specific spinal populations. Potential OC factor targets that could mediate indirect repressive regulations include microRNAs (miRs), as OC factors have been shown to stimulate miR-122 in hepatic cells^49^. However, miR contributions to spinal cord development remain poorly described. Third, OC factors may associate with cell-type specific cofactors or transcriptional complexes to differentially regulate expression in distinct cell populations. In hepatic cells, OC factors interact with the CBP, p300 and PGC-1 cofactors to stimulate the expression of specific target genes^48,50^. Similarly, we recently demonstrated that CBP and p300 cofactors contribute to the regulation of *Isl1* expression by OC factors^51^. However, association of OC factors with transcriptional repressors remains to be demonstrated. This highlights the urgent need to identify direct transcriptional targets and molecular partners of OC factors in specific spinal neuron populations to better understand their contribution to spinal cord development.

Inactivation of the *Nkx6.2* gene in mice or mutations within the orthologous gene in humans result respectively in motor deficits that have been attributed previously to defective central myelination^23,52,53^. Although we cannot formally exclude contributions from such myelin-associated pathophysiology, perturbed embryonic expression of *Nkx6.2* in V2a INs and in LMCl MNs, which are part of spinal motor circuits including the locomotor network, prompted us to evaluate the effects of the absence of Nkx6.2 specifically on locomotor behavior in *Nkx6.2*-null adults and neonates, and on FL in neonates. Adult locomotion showed increased gait frequency and altered paw-substrate contact patterns, and neonatal locomotion (swimming) showed increased cycle frequency and defective alternation, suggesting potential perturbations of the locomotor network. Recording of FL in isolated neonatal *Nkx6.2*-null spinal cord preparations revealed no obvious perturbation of pattern, but a change in the frequency profile characterized by a shift from the control bimodal frequency distribution to a distribution dominated by a single intermediate frequency mode. In the locomotor CPG, V2a INs provide left/right alternation at high frequency^54^ whereas their target V0 INs are divided into inhibitory or excitatory subsets that ensure left/right alternation at low or high locomotor speeds, respectively^55^. Since we find that the absence of Nkx6.2 perturbs the development of V2a INs, it is possible that this also perturbs their connectivity in a way that alters locomotor network output. Our observations in the current study are unlikely to be secondary to the absence of *Nkx6.2* expression in oligodendrocytes, even though myelination of cervical spinal MN axons and central vestibular projections commences perinatally in mice^41,56^. Comparison of the locomotor defects in *Nkx6.2* and in constitutive or MN-specific conditional *Oc* mutant animals unfortunately can not be conducted because constitutive^24^ and conditional (data not shown) *Oc* mutants die at birth for reasons that remain to be determined. Similarly, identifying the reasons why the RF portion of the adult quadriceps of *Nkx6.2*-null adults shows an increase in the number of fatiguable large diameter fibers at the expense of fatigue-resistant small diameter fibers will also require further investigation.

In spinal neuronal progenitors, Nkx6.2 function overlaps with that of Nkx6.1 when it comes to ventral patterning of the spinal cord^35^. However, Nkx6.2 is additionally expressed later in V2a INs and in a pool of LMCl MNs (ref. ^38^ and the present study). Since the only progenitors that express *Nkx6.2* are the V1 progenitors^37^, expression in the postmitotic V2a INs and LMCl MNs is not merely a carry-over from progenitor expression but rather a secondarily regulated pattern. That Nkx6.2 is actively regulated in two postmitotic spinal neuron populations suggests a possible role in controlling their developmental programs. Our phenotypic study of *Nkx6.2*-null embryos suggests that Nkx6.2 regulates distinct biological processes in MNs and ventral INs. In *Nkx6.2*-null embryos, the number of LMCl MNs was normal at e12.5 but depleted at e14.5, indicating that Nkx6.2 is required for the survival of some LMCl MNs. The most immediate explanation is that the missing MNs correspond to the RF motor pool that normally expresses *Nkx6.2*. However, several observations argue against this simple interpretation. First, β-galactosidase-positive MNs, i.e. the MNs in which the Nkx6.2 regulating sequences are normally activated, are found in the ventral portion of the LMCl in *Nkx6.2*-null embryos, in an area that does not contain RF MNs. Second, motor projections towards the RF are present and seem unaltered in the *Nkx6.2* mutant^38^. Third, the density of neuromuscular junctions in the RF is normal in the *Nkx6.2* mutant (data not shown). Taken together, these data suggest that RF MNs are not significantly affected by the absence of Nkx6.2. In an attempt to identify the MN subset that could be missing in the *Nkx6.2* mutant, we evaluated the size of the Nkx6.1 + or Er81 + motor pools but found that these MNs were also present in normal numbers. Thus, the identity of the LMCl cells that are missing in *Nkx6.2* mutants and the apparently non-cell autonomous mechanisms whereby Nkx6.2 promotes their survival remain to be determined.

In contrast to LMCl MNs, the number of V2a INs is largely unaffected by the absence of Nkx6.2, except for a modest reduction in the thoracic spinal cord. However, the spatial distribution of the V2a INs was abnormal. This is consistent with the downregulation of *Nkx6.2* expression in V2a INs and the perturbation of V2a IN distribution recently reported in constitutive *Oc* mutant embryos^13^. These data suggest that Nkx6.2 contributes to regulate V2a IN distribution downstream of the OC factors. To our knowledge, Nkx6.2 has not previously been reported to regulate neuronal localization, and the mechanisms whereby it does here are not known. Similarly, OC factors and Pou2f2 have recently been shown to regulate the distribution of V2a and V2b INs but their downstream targets in this process remain unknown. A genome-wide systematic study of the transcriptional targets of the three OC transcription factors would be required to understand the downstream genetic programs and mechanisms regulating the distribution of ventral INs during spinal cord development.

## Supplementary information

Dataset 1.

## Data Availability

All data generated or analysed during this study are included in this published article (and its Supplementary Information Files) or available upon request.
