# Author Correction: Onecut-dependent Nkx6.2 transcription factor expression is required for proper formation and activity of spinal locomotor circuits

**DOI:** 10.1038/s41598-020-70339-w

**Published:** 2020-08-06

**Authors:** Mathilde Toch, Audrey Harris, Olivier Schakman, Elena Kondratskaya, Jean-Luc Boulland, Nicolas Dauguet, Stéphanie Debrulle, Charlotte Baudouin, Maria Hidalgo-Figueroa, Xiuqian Mu, Alexander Gow, Joel C. Glover, Fadel Tissir, Frédéric Clotman

**Affiliations:** 1 Université catholique de Louvain, Institute of Neuroscience, Laboratory of Neural Differentiation, Brussels, Belgium; 2 Université catholique de Louvain, Institute of Neuroscience, Laboratory of Cell Physiology, Brussels, Belgium; 3 Laboratory for Neural Development and Optical Recording (NDEVOR), Section for Physiology, Department of Molecular Medicine, Institute of Basic Medical Sciences, University of Oslo, Oslo, Norway; 4 Norwegian Center for Stem Cell Research, Department of Immunology and Transfusion Medicine, Oslo University Hospital, Oslo, Norway; 5 Université catholique de Louvain, de Duve Institute, Flow cytometry and cell sorting facility (CYTF), Brussels, Belgium; 6 Department of Ophthalmology/Ross Eye Institute, New York State Center of Excellence in Bioinformatics and Life Sciences, University at Buffalo, Buffalo, NY 14203 USA; 7 Wayne State University, Center for Molecular Medicine and Genetics, Carman and Ann Adams Department of Pediatrics, Department of Neurology, Detroit, Michigan USA; 8 Université Catholique de Louvain, Institute of Neuroscience, Laboratory of Developmental Neurobiology, Brussels, Belgium; 9 CIBER de Salud Mental (CIBERSAM), Madrid, Spain; 10 University of Cadiz, Cadiz, Spain

Correction to: *Scientific Reports*10.1038/s41598-020-57945-4, published online 22 January 2020

Xiuqian Mu was omitted from the author list in the original version of this Article. This has been corrected in the PDF and HTML versions of the Article, and in the accompanying Supplementary Information file.

The Author Contributions section now reads:

M.T., A.H., E.K., J.-L.B., J.C.G., N.D., F.T. and F.C. designed the experiments. M.H.-F., X.M. and A.G. provided mice and contributed to initial discussions. M.T., A.H., O.S., E.K., J.-L.B., N.D., S.D. and C.B. performed the experiments, M.T., A.H., O.S., E.K., and J.-L.B. contributed to data analyses and all the authors discussed the data for interpretation. M.T. and F.C. wrote the initial draft of the manuscript, and revisions were made by J.C.G., J.-L.B., E.K., A.G., M.T. and F.C.

In addition, the Acknowledgements section now reads:

We thank members of the NEDI lab for material, technical support and discussions. This work was supported by grants from the "Fonds spéciaux de recherche" (FSR) of the Université catholique de Louvain, by the "Actions de Recherche Concertées (ARC)" #17/22-079 of the "Direction générale de l’Enseignement non obligatoire et de la Recherche scientifique – Direction de la Recherche scientifique – Communauté française de Belgique" and granted by the "Académie universitaire ‘Louvain’", by a "Projet de recherche (PDR)" #T.0117.13 and an "Equipement (EQP)" funding #U.N027.14 of the Fonds de la Recherche Scientifique (F.R.S.-FNRS) and by the Association Belge contre les Maladies neuro-Musculaires (ABMM, Belgium) to F.C., by South-East Norway Health Authority grant no. 2014119 to J.-L.B., by Norwegian Research Council grant no. 230000/F20 and EU-funded ERA-NET Neuron grant (CERMOD) to J.C.G., by a grant (R01EY029705) from the National Institutes of Health/National Eye Institute to X.M. and by NINDS in the National Institutes of Health, grant no. NS102678 to A.G. A.H., S.D. and C.B. held FRIA grants and M.H.-F. held a Research Associate grant from the F.R.S.-FNRS. F.C. and F.T. are Senior Research Associate and Research Director of the F.R.S.-FNRS, respectively.

