# Supplementary material for: Onecut-dependent Nkx6.2 transcription factor expression is required for proper formation and activity of spinal locomotor circuits
Source: Sci Rep. 2020 Jan 22;10:996. doi: 10.1038/s41598-020-57945-4 (PMC6976625; doi:10.1038/s41598-020-57945-4)
Supplement: Supplementary file 1 — Dataset 1. [file 41598_2020_57945_MOESM1_ESM.pdf]

# Onecut-dependent Nkx6.2 transcription factor expression is required for proper formation and activity of spinal locomotor circuits

Mathilde Toch[1], Audrey Harris[1], Olivier Schakman[2], Elena Kondratskaya[3], Jean-Luc Boulland[4], Nicolas Dauguet[5], Stéphanie Debrulle[1], Charlotte Baudouin[1], Maria Hidalgo-Figueroa[1,9,10], Xiuqian Mu[6], Alexander Gow[7], Joel C. Glover[3,4], Fadel Tissir[8] and Frédéric Clotman[1,*]

*** Corresponding author email**: frederic.clotman@uclouvain.be

## Supplemental table and figure legends

**Supplemental Table 1. RNA-seq comparison of control and conditional *Oc*-deficient motor neurons**. The differential expression of selected candidates is shown as fold change in cdKO vs. control MNs. Adjpval: adjusted p-value. *The sequences detected for *Oc2* correspond to truncated transcripts encompassing the end of exon 1 and exon 2 that are generated in *Oc2-/-* tissues and do not generate any functional peptide [25].

**Supplemental Fig.1. Vestibulospinal reflex test in neonatal mice**. The vestibulospinal reflex test measures compensatory extension of hindlimbs after a rapid (<200 msec) body and head rotation that activates the vestibular apparatus. (**A**) Newborn mice are immobilized in a Teflon holder at the level of the trunk, leaving the limbs free to move. The 90° rotation of the body and head generates a reflex extension of the hindlimb on the side to which the head is rotated (arrows). (**B**) Comparison of the position of the hindlimb at the start and at the end of the rotation (distance PCT, magenta line). (**C**) PCT quantification after vestibulospinal reflex test in heterozygous control (n=11) and *Nkx6.2* mutant (n=13) newborn mice showed no difference in compensatory extension.

**Supplemental Fig.2. Portions of quadriceps muscle**. Hemalun/eosin labelings of transverse section from the quadriceps muscle. The quadriceps muscle is divided into four portions: *Rectus femoris* (RF), *Vastus medialis* (VM), *Vastus intermedius* (VI) and *Vastus lateralis* (VL).

**Supplemental Fig.3. Absence of *Nkx6.2* does not alter the formation of Nkx6.1+ or Er81+ motoneuron pools.** Immunostaining of transverse spinal cord sections from $Nkx6.2^{+/-}$ (heterozygous control) or $Nkx6.2^{-/-}$ mutant embryos at e14.5. The number of MNs in the Nkx6.1+ (**A, C, E**) or Er81+ (**B, D, E**) motoneuron pools was not affected by the absence of Nkx6.2. n=3. Scale bar = 50 µm.

| Gene | Symbol | Fold change | pvalue | adjpvalue |
|---|---|---|---|---|
| Tachykinin precursor 1 | Tac1 | 49.10 | 2,01E-12 | 1,06E-08 |
| Paired Related Homeobox-Like 1 | Prrxl1 | 6.37 | 1.48E-06 | 0.0026 |
| Small Integral Memrabne Protein 18 | Smim18 | 5.48 | 0.0007 | 0.3281 |
| Short Stature Homeobox 2 | Shox2 | 4.98 | 0.0027 | 0.5210 |
| LIM Domain Binding 2 | Ldb2 | 4.36 | 1,69E-05 | 0.0177 |
| Stabilizer of Axonemal Microtubules 2 | Saxo2 | 3.84 | 0.0020 | 0.4738 |
| Nk6 homeobox 2 | Nkx6.2 | 1.32 | 0.3656 | 0.9999 |
| Onecut 2 | Oc2 | 1.21 * | 0.6471 | 0.9999 |
| Plexin Domain Containing 2 | Plxdc2 | 0.43 | 0.0027 | 0.5242 |
| Fibroblast Growth Factor 18 | Fgf18 | 0.40 | 0.0014 | 0.4085 |
| GDNF Family Receptor Alpha 3 | Gfra3 | 0.33 | 8.38E-05 | 0.0735 |
| Basic Helix-Loop-Helix Family Member E23 | Bhlhe23 | 0.21 | 2.38E-08 | 7.52E-05 |
| Semaphorin 3D | Sema3d | 0.21 | 2.28E-06 | 0.0035 |
| Onecut 1 | Oc1 | 0.20 | 1.63E-07 | 0.0003 |
| Iroquois Homeobox 6 | Irx6 | 0.16 | 2.45E-06 | 0.0035 |
| POU Class 6 Homeobox 2 | Pou6f2 | 0.11 | 0.0002 | 0.1313 |
| Onecut 3 | Oc3 | 0.005 | 1.62E-12 | 1.06E-08 |

Supplemental table 1 – Toch M. et al.

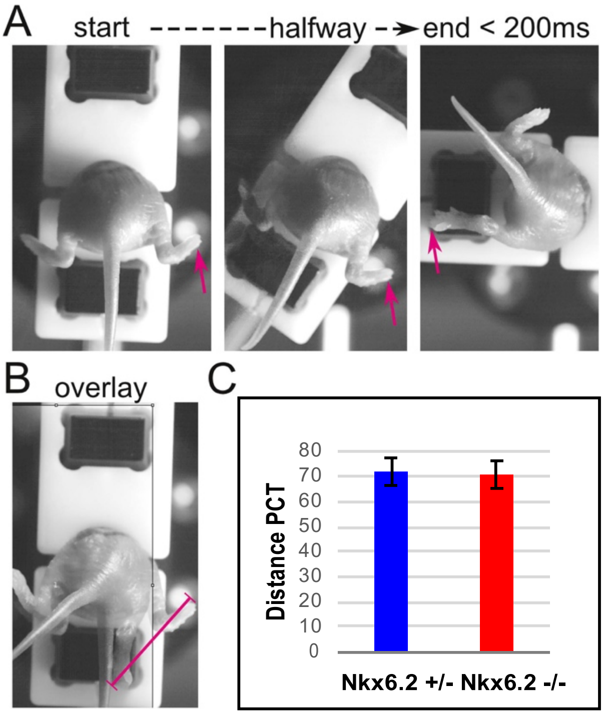


Supplemental figure 1 – Toch M. et al.

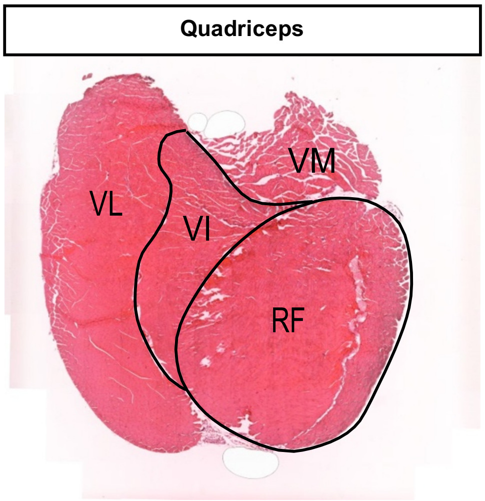


Supplemental figure 2 – Toch M. et al.

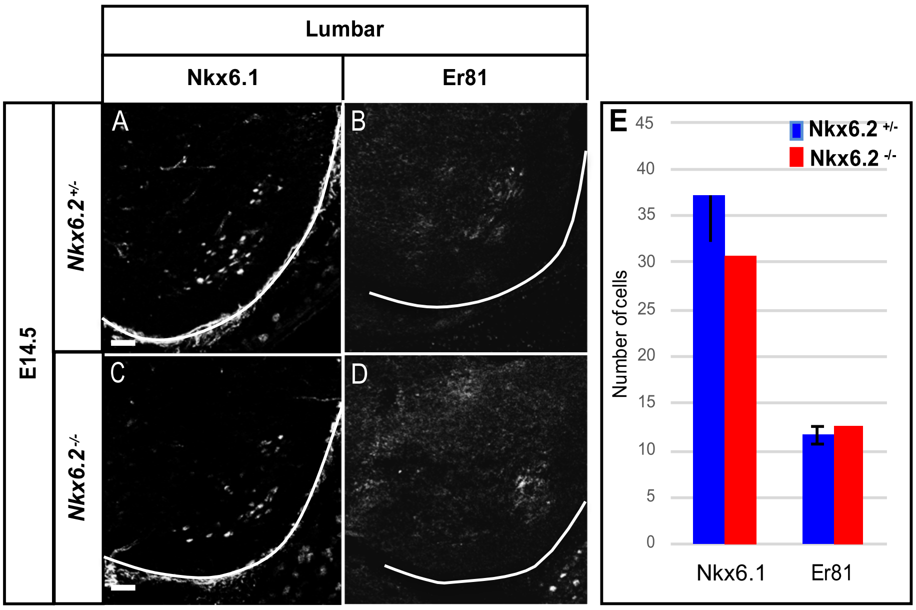


Supplemental figure 3 – Toch M. et al.
